# MRE11 as a Predictive Biomarker of Outcome After Radiation Therapy in Bladder Cancer

**DOI:** 10.1016/j.ijrobp.2019.03.015

**Published:** 2019-07-15

**Authors:** Alexandra K. Walker, Katalin Karaszi, Helen Valentine, Victoria Y. Strauss, Ananya Choudhury, Shaun McGill, Kaisheng Wen, Michael D. Brown, Vijay Ramani, Selina Bhattarai, Mark T.W. Teo, Lingjian Yang, Kevin A. Myers, Nayneeta Deshmukh, Helen Denley, Lisa Browning, Sharon B. Love, Gopa Iyer, Noel W. Clarke, Emma Hall, Robert Huddart, Nicholas D. James, Peter J. Hoskin, Catharine M.L. West, Anne E. Kiltie

**Affiliations:** ∗ CRUK/MRC Oxford Institute for Radiation Oncology, University of Oxford, Oxford, United Kingdom; † Translational Radiobiology Group, Division of Cancer Sciences, Christie Hospital NHS Foundation Trust, Manchester Academic Health Science Centre, University of Manchester, Manchester, United Kingdom; ‡ Centre for Statistics in Medicine, Nuffield Department of Orthopaedics, Rheumatology and Musculoskeletal Diseases, Botnar Research Centre, University of Oxford, Oxford, United Kingdom; § School of Cancer Sciences, University of Birmingham, Birmingham, United Kingdom; ‖ Genito Urinary Cancer Research Group, Division of Cancer Sciences, Faculty of Biology, Medicine & Health, University of Manchester, Manchester, United Kingdom; ¶ Department of Urology, The Christie NHS Foundation Trust, Manchester, United Kingdom; # Department of Histopathology, Leeds Teaching Hospitals NHS Trust, Leeds, United Kingdom; ∗∗ Leeds Cancer Centre, St James's University Hospital, Leeds, United Kingdom; †† Experimental Cancer Medicine Centre, Department of Oncology, University of Oxford, Oxford, United Kingdom; ‡‡ Department of Cellular Pathology, Manchester University Foundation Trust, Manchester, United Kingdom; §§ Department of Cellular Pathology, Oxford University Hospitals NHS Foundation Trust, John Radcliffe Hospital, Oxford, United Kingdom; ‖‖ NIHR Oxford Biomedical Research Centre, Oxford, United Kingdom; ¶¶ Weill Cornell Medical College, Cornell University, New York, New York; ## Clinical Trials and Statistics Unit, Institute of Cancer Research, London, United Kingdom; ∗∗∗ Academic Uro-Oncology Unit, The Royal Marsden NHS Foundation Trust, Sutton, London, United Kingdom; The Institute of Cancer Research, London, United Kingdom; ††† Cancer Centre, Mount Vernon Hospital, Northwood, Middlesex, United Kingdom; ‡‡‡ Manchester Cancer Research Centre, University of Manchester, Manchester, United Kingdom

## Abstract

**Purpose:**

Organ-confined muscle-invasive bladder cancer is treated with cystectomy or bladder preservation techniques, including radiation therapy. There are currently no biomarkers to inform management decisions and aid patient choice. Previously we showed high levels of MRE11 protein, assessed by immunohistochemistry (IHC), predicted outcome after radiation therapy, but not cystectomy. Therefore, we sought to develop the MRE11 IHC assay for clinical use and define its relationship to clinical outcome in samples from 2 major clinical trials.

**Methods and Materials:**

Samples from the BCON and BC2001 randomized controlled trials and a cystectomy cohort were stained using automated IHC methods and scored for MRE11 in 3 centers in the United Kingdom.

**Results:**

Despite step-wise creation of scoring cards and standard operating procedures for staining and interpretation, there was poor intercenter scoring agreement (kappa, 0.32; 95% confidence interval, 0.17-0.47). No significant associations between MRE11 scores and cause-specific survival were identified in BCON (n = 132) and BC2001 (n = 221) samples. Reoptimized staining improved agreement between scores from BCON tissue microarrays (n = 116), but MRE11 expression was not prognostic for cause-specific survival.

**Conclusions:**

Manual IHC scoring of MRE11 was not validated as a reproducible biomarker of radiation-based bladder preservation success. There is a need for automated quantitative methods or a reassessment of how DNA-damage response relates to clinical outcomes.

**Summary**

We attempted to develop an MRE11 immunohistochemistry assay to appropriate standards for clinical use and to define the relationship of the biomarker to clinical outcome in a prospective analysis of retrospective tissue from 2 randomized trials. Staining was reproducible across centers, but scoring was less so; thus, we could not validate MRE11 as a robust, reproducible predictive biomarker of radiation therapy response. Our study demonstrates the challenges involved in developing a robust immunohistochemistry-based protein biomarker.

## Introduction

Nonmetastatic muscle-invasive bladder cancer (MIBC) can be treated with curative intent by either cystectomy or bladder preservation techniques, including radiation therapy (RT) alone or with a radiosensitizing agent if tolerated^1^, ^2^; neoadjuvant cisplatin-based chemotherapy is often used for suitable patients. These approaches have a 40% to 60% cause-specific survival (CSS) rate at 3 years.^3^, ^4^ With no randomized data available, treatment is currently based on patient choice after discussion with a urologist, oncologist, and nurse specialist.^5^ To date there are no validated biomarkers to predict the likely patient benefit from either approach.^6^

We previously showed using immunohistochemistry (IHC) on pretreatment transurethral resection of bladder tumor (TURBT) specimens that high levels of MRE11, a DNA-damage signaling protein, predicted outcome after radical RT for MIBC in 2 independent cohorts, but not after cystectomy.^7^ Our results were subsequently independently validated in a Danish/German study.^8^

The aims of the present study were (1) to evaluate the ability to standardize the MRE11 IHC assay and its scoring methodology across multiple centers in the United Kingdom, thus developing it to appropriate standards for clinical use (final stage of biomarker discovery and assay development phase)^9^ and (2) to again validate its correlation with outcome in 2 of the largest and most important recent randomized trials of bladder preservation in MIBC (prospective analysis of retrospective tissue collections; first stage of biomarker qualification phase). Reporting Recommendations for Tumor Marker Prognostic Studies guidelines were followed.^9^

## Methods and Materials

Ethical approval was obtained from the National Research Ethics services in Manchester (project 09/H1013/24 and 10_NOCL_O1), Oxford (09/H0606/5), and Birmingham (REC 00/8/75). All trial patients consented to use of their tissue and data for research.

### Study populations

Patients in the UK multicenter randomized controlled trials BCON and BC2001 were given RT as 64 Gy in 32 fractions over 6.5 weeks or 55 Gy in 20 fractions over 20 weeks. BCON patients were randomized between RT alone or RT with carbogen and nicotinamide (132 whole mount and 116 tissue microarray [TMA] samples available).^1^ BC2001 (CRUK/01/004) patients were randomized to RT alone or RT with 5-fluorouracil and mitomycin C (317 samples; split into test [n = 154] and validation [n = 163] cohorts).^2^ One hundred samples were obtained from a cystectomy series in Manchester.^10^

### Materials

Pretreatment formalin-fixed paraffin-embedded TURBT samples (Table E1; available online at https://doi.org/10.1016/j.ijrobp.2019.03.015) were available for the trial and cystectomy cohorts. For BCON, whole formalin-fixed paraffin-embedded blocks and 1 mm core TMAs from invasive areas (constructed in Manchester in 2011) were used. Two in-house “BIDD” TMAs containing 0.6 mm diameter cores were created for assay development in Oxford (Material E1; available online at https://doi.org/10.1016/j.ijrobp.2019.03.015). Sections (4 μm thick) were cut and stored at 4°C before use, with IHC performed no later than 1 month after cutting. A consultant uropathologist outlined areas of urothelial carcinoma invading the lamina propria (T1) and/or muscularis propria (T2) on hematoxylin and eosin– or MRE11-stained sections. Tumors with divergent differentiation within the invasive component were regarded as invasive urothelial carcinoma.

### MRE11 immunohistochemistry

After pilot work, a standardized operating procedure (SOP) was produced for MRE11 IHC using a Leica BOND-MAXautostainer (Leica Microsystems GmbH, Wetzlar, Germany) according to the manufacturer's instructions in Oxford, Manchester, and Birmingham (see Materials E1; available online at https://doi.org/10.1016/j.ijrobp.2019.03.015). Slides were dewaxed in Bond Dewax solution (AR9222, Leica Microsystems) and rehydrated through graded ethanol and distilled water. Tissue sections were washed using Bond Wash solution (AR9590, Leica Microsystems). Endogenous peroxidases were blocked using peroxidase block solution for 5 minutes, followed by antigen retrieval at pH 6 using Epitope Retrieval 1 solution (AR9961, Leica Microsystems) for 20 minutes at 100°C. Slides were then incubated with mouse monoclonal anti-MRE11 antibody (1:3,000, Abcam plc, Cambridge, UK, ab214, 1 mg/ml) for 15 minutes at room temperature. Primary antibody binding to tissue sections was visualized using a biotin-free Bond polymer refine detection system (DS9800, Leica Microsystems). After postprimary amplification for 8 minutes and detection with polymer for 8 minutes using 3,3'-diaminobenzidine for 10 minutes, slides were counterstained with hematoxylin for 1 minute.

Six control slides were included in every 30-slide run. Negative control samples were stained using a mouse monoclonal immunoglobulin (Dako, Glostrup, Denmark, X0931, 100 mg/L) diluted to the same concentration as the MRE11 antibody; samples consisted of 2 patient samples from the cohort being stained and 1 slide from the BIDD TMA and a commercial sample. Positive controls consisted of sections from 3 of the commercial bladder tumor samples and a BIDD TMA section stained with the MRE11 antibody.

During the study, the automated IHC was improved by adding a 30 minute 10% bovine serum albumin (BSA) preprimary antibody protein blocking step, increasing the primary antibody dilution from 1:3000 in 1% BSA to 1:6000 in 10% BSA and reducing the primary antibody incubation time from 15 minutes to 8 minutes. After reoptimization, IHC was repeated on the BCON TMAs (final SOP in Material E1; available online at https://doi.org/10.1016/j.ijrobp.2019.03.015).

In Leeds, BCON samples were stained using an Autostainer Link 48 instrument (Dako, Inc) and an EnVision FLEX+, Mouse, High pH kit (Dako, K8002). Slides were deparaffinized and pretreated in the automated Dako PT Link system using heated Envision Flex target high pH retrieval solution. Endogenous peroxidases were blocked using Flex Peroxidase Block for 5 minutes. Slides were incubated with mouse monoclonal anti-MRE11 antibody (1:3,000, Abcam plc, ab214, 1 mg/mL) for 30 minutes at room temperature. After applying labelled polymer Flex/horseradish peroxidase for 20 minutes, the staining was visualized using Flex DAB+ substrate chromogen for 2 × 5 minutes, and slides were counterstained with hematoxylin.

### MRE11 scoring

**Fig. E1:**
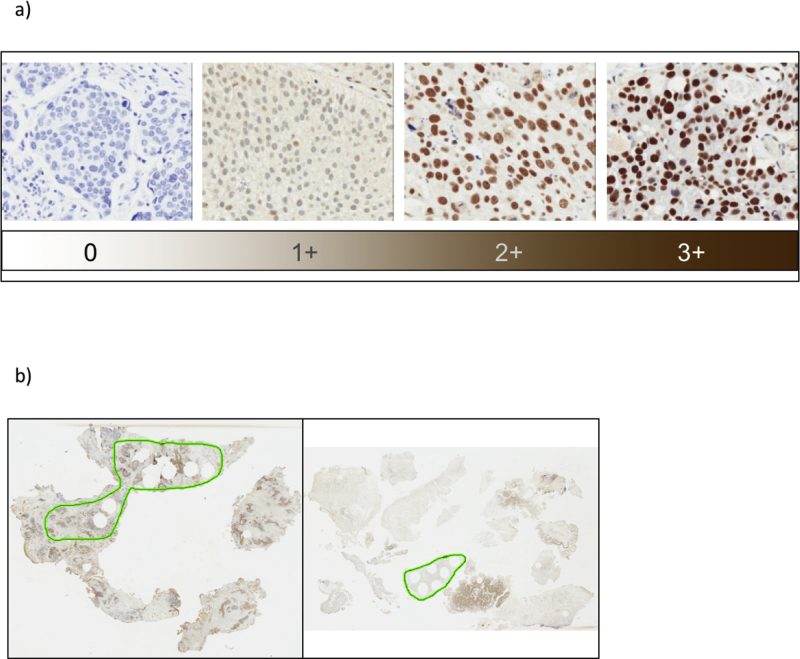


Slides were scanned using an Aperio ScanScope CS scanner (Leica Microsystems) at 40× magnification and visualized by ImageScope Viewer. Scoring similar to that we have described elsewhere^7^ was undertaken after training, with guidance sought on IHC interpretation from a histopathologist for challenging cases (details in Material E1; available online at https://doi.org/10.1016/j.ijrobp.2019.03.015). Briefly, 6 to 10 images (containing at least 100 cells) were taken from random fields within the invasive areas. Surface papillary tumors and carcinoma in situ were not scored. Care was taken to avoid taking images from areas distorted or damaged by diathermy or crush artefact and from necrotic areas, reducing the potential of including cells with unreliable immunostaining. Tumor nuclear MRE11 staining intensity was graded as 0 to 3+ (Fig. E1a; available online at https://doi.org/10.1016/j.ijrobp.2019.03.015) by 2 to 3 independent scorers within each center using a guide composed of 30 images. The modal intensity for each of the 6 to 10 images was determined and an overall modal intensity score assigned to each case. Comparison was then made of results from individual scorers, with differences highlighted and a consensus reached for each center. Percentage positivity was determined by either (initially) counting 100 cells per image using ImageJ software (Bethesda, MD)^11^ or using a second standardization scoring guide with 30 images to estimate the percentage positivity. The mean percentage of positive cells was multiplied by the modal intensity to give a semiquantitative H-score (0-300, Table E2; available online at https://doi.org/10.1016/j.ijrobp.2019.03.015).

### Statistical analysis

Analyses were conducted independently via STATA in each cohort, with a 25% cut off used to allow comparison with previous publications. Within each center, for a cohort, the interrater agreement of MRE11 intensity scores was assessed using the weighted kappa statistic via STATA kappaetc packages. The reliability of the percentage of positive scores was studied using intraclass correlation coefficient (ICC). A higher value indicates a better agreement between scorers.^12^ Between centers, the reliability of MRE11 intensity and percentage positive scores were assessed by the kappa statistic and the ICC, respectively. Associations between MRE11 H-score (≤25th percentile and >25th percentile) and bladder CSS were analyzed using Kaplan-Meier graphs with log-rank tests. In the BCON TMA cohort, a subgroup analysis was conducted for RT patients only. Hazard ratios were generated for MRE11 H-score >25th percentile using Cox regression with adjustment for treatment, stage, grade, completion of TURBT, pretreatment hemoglobin level, and number of RT fractions.

## Results

**Fig. 1:**
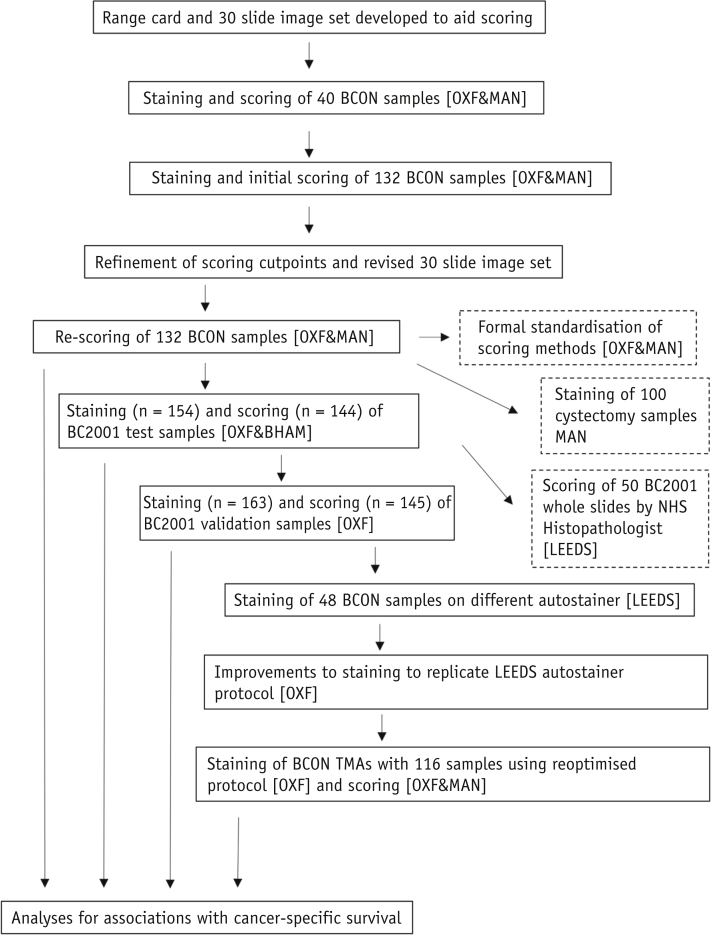
Schematic of experiments undertaken. *Abbreviations:* BHAM = Birmingham, MAN = Manchester, OXF = Oxford.

A schematic of the study design is presented in Figure 1.

### Staining and scoring reliability

The MRE11 assay was developed in Oxford (see original standard operating procedure). Staining was highly reproducible between runs. A working dilution of 1:3000 was agreed by A.K., K.K., and K.M. A range card was created from the MIBC samples from Oxford to represent 0, 1+, 2+, and 3+ intensity scores for tumor nuclear MRE11 expression (Fig. E1; available online at https://doi.org/10.1016/j.ijrobp.2019.03.015). A 30-sample PowerPoint slide deck (Microsoft, Redmond, WA) was then developed for subsequent scoring by A.K., K.K., A.C., and M.T. (the latter 2 were experienced in manual scoring).^7^ Blinded scoring resulted in concordance among scores from 4 observers in 57% of the 30 cases (87% agreement for 3 scorers). After discussion, agreement was achieved for all samples.

**Fig. 2:**
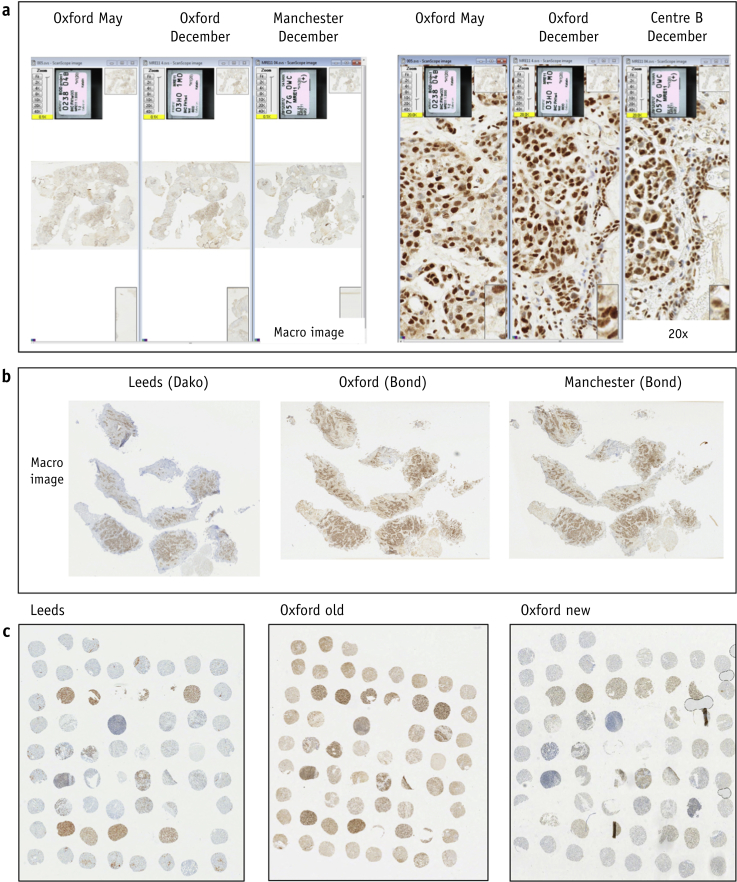
Comparison of MRE11 staining between (a) initial (May) Oxford and subsequent (December) Oxford and Manchester staining of BCON samples; (b) Leeds, Oxford, and Manchester staining of BCON samples; (c) reoptimized staining (Oxford new) on BIDD tissue microarray compared to Leeds staining and original Oxford staining (Oxford old).

**Fig. E2:**
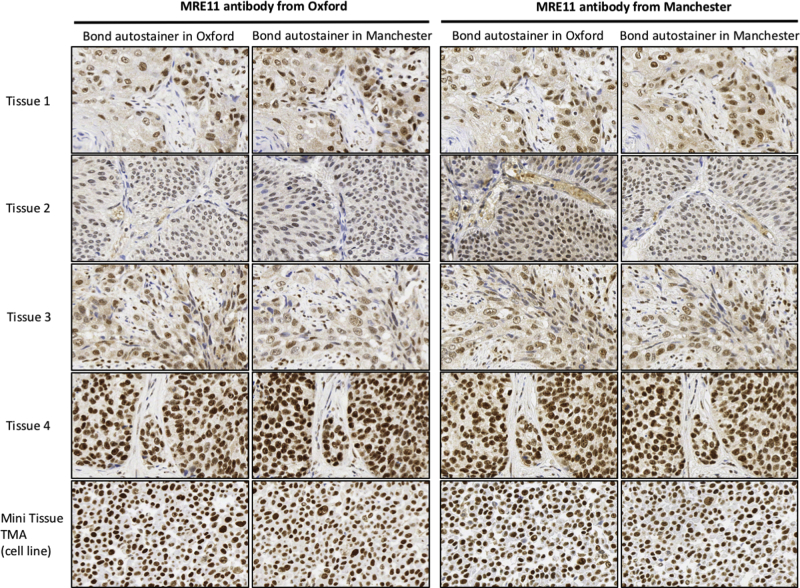


Initially 40 BCON slides (parallel whole sections) were stained and scored in Manchester and Oxford, and 7 months later a further 132 slides were stained and scored. Staining was similar between centers and antibody aliquots (Table 1 and Fig. E2; available online at https://doi.org/10.1016/j.ijrobp.2019.03.015) and across time (Fig. 2a). However, scores were higher in Oxford, with 87 of the 132 samples (66%) scored as 3+ versus 50 (38%) in Manchester (Table 2). The data equated to H-score 25th percentile cut-points of 175.8 for Manchester and 204.1 for Oxford; in the test and validation cohorts of Choudhury et al,^7^ these were 130 and 76, respectively.

**Table 1:** Intra- and intercenter scoring agreement

Comparison	Initial staining		New staining	
	Kappa (95% CI) on intensity	ICC (95% CI) on % positive cells	Kappa (95% CI) on intensity	ICC (95% CI) on % positive cells
BCON (n = 132)				
Intracenter: Oxford scorers	0.63 (0.52-0.73)	NA∗	0.59 (0.49-0.68)	0.90 (0.88-0.93)
Intracenter: Manchester scorers	0.75 (0.64-0.85)	NA∗	0.95 (0.91-0.99)	0.98 (0.98-0.99)
Intercenter: Oxford-Manchester	0.32 (0.17-0.47)	0.73 (0.64-0.82)	0.55 (0.41-0.70)	0.91 (0.88-0.94)
BC2001				
Testing (n = 144)	0.66 (0.58-0.74)	0.95 (0.93-0.96)	NA	NA
Validation (n = 145)	0.72 (0.62-0.83)	NA†	NA	NA

*Abbreviations:* CI = confidence interval; ICC = intraclass correlation coefficient; NA= not applicable.

∗ % positive cells were scored by 1 scorer.

† Not possible to calculate as there was very little variation in slides for 1 scorer.

**Table 2:** Comparison of intensity scores for BCON and BC2001 cohorts

Intensity	BCON original		BCON revised		BC2001 test cohort		BC2001 validation cohort
	Oxford	Manchester	Oxford	Manchester	Oxford	Birmingham	Oxford
0	0	0	0	0	0	0	0
1+	4	18	11	22	19	3	8
2+	24	48	62	77	81	33	81
3+	87	50	42	17	44	102	56
Not good	17	16	17	16	10	16	18
Total	132	132	132	132	154	154	163

BCON uses original scoring cut points of Oxford versus Manchester and revised scoring cut points of Oxford versus Manchester. The BC2001 test cohort is Oxford versus Birmingham (K.W.), and the BC2001 validation cohort is Oxford.

We concluded that automated staining and improved imaging resulted in higher scores than the manual methods previously used. The 1+/2+ and 2+/3+ cut-point boundaries were then redefined and a revised 30-slide template produced. Rescoring reduced the number of 3+ scores to 42 of 132 (32%) for Oxford and 17 of 132 (13%) for Manchester (Table 2), with respective median H-scores reduced from 197 to 148 and 195 to 149 (Table E2; available online at https://doi.org/10.1016/j.ijrobp.2019.03.015).

Before the data were linked with survival outcomes, a more formal assessment of standardization of scoring methods was undertaken. Twenty randomly selected Oxford images were scored by 2 or 3 Manchester observers (C.W., H.V., A.C.), with consensus reached and comparison made with Oxford scores; a similar procedure was performed for 20 randomly selected Manchester images scored by 3 Oxford observers (A.K., K.K., A.W.). Manchester scores tended to be lower than Oxford scores. Three of 20 Manchester and 3 of 20 Oxford cases were discordant, although consensus was reached in all but 1 of the latter (Table E3; available online at https://doi.org/10.1016/j.ijrobp.2019.03.015).

One hundred fifty-four BC2001 test slides were stained and 144 scored in both Oxford and Birmingham. The Birmingham observer scored 102 (66%) samples as 3+ compared with 44 (29%) in Oxford. In Oxford, a further 163 BC2001 validation slides were stained and imaged (by K.K.), and staining intensity and estimated positive percentage were scored in 145 slides independently by 2 observers (A.K. and K.K.) and consensus scores reached.

### Associations with cancer-specific survival

Analyses carried out using data generated with the initial staining procedure in BCON samples showed no significant associations between MRE11 expression and CSS (Fig. E4a and E4b; available online at https://doi.org/10.1016/j.ijrobp.2019.03.015). Analysis of evaluable samples from 221 training and validation BC2001 patients by Oxford confirmed the lack of significant association between MRE11 expression and CSS (log rank test *P* = .97; Fig. E4c, available online at https://doi.org/10.1016/j.ijrobp.2019.03.015). Analysis of 99 patients in the cystectomy cohort showed a lack of significant association between MRE11 expression and CSS (log rank test *P* = .19; Fig. E4d, available online at https://doi.org/10.1016/j.ijrobp.2019.03.015).

### Refinement of the assay

**Fig. E3:**
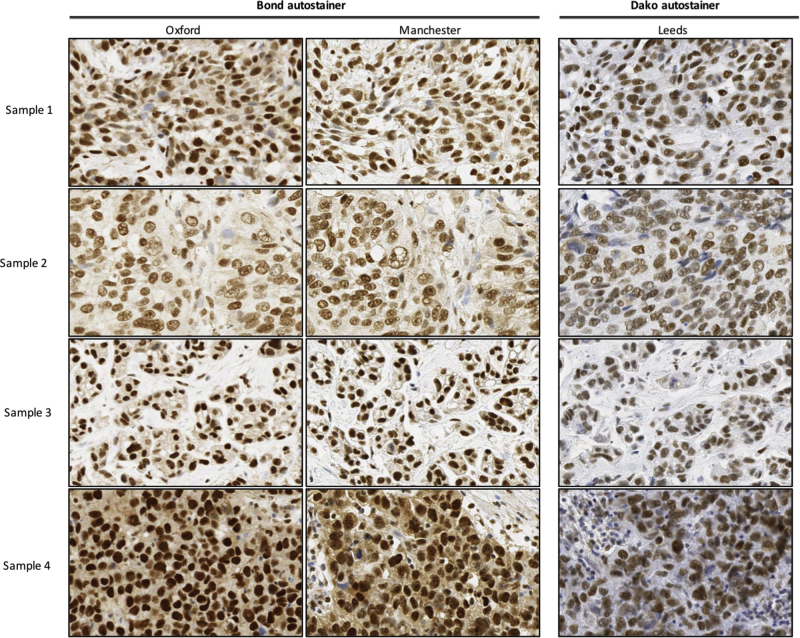


**Fig. E4:**
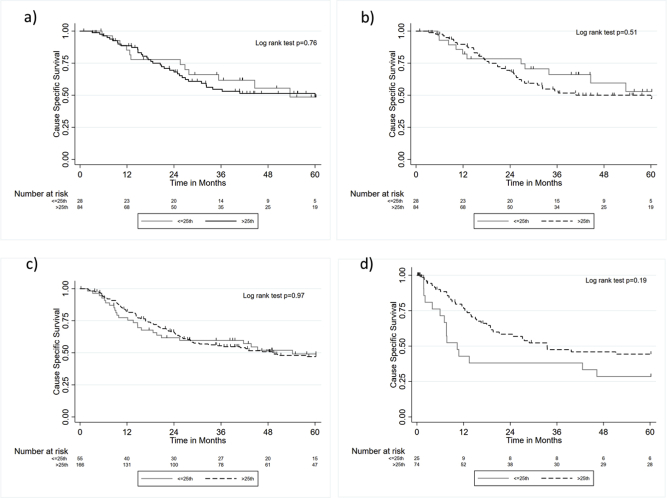


A subset of BCON samples (n = 48) stained in the Leeds Pathology Department (Fig. 2b and 2c Fig. E3, available online at https://doi.org/10.1016/j.ijrobp.2019.03.015) showed reduced nonspecific background staining compared with samples stained in Oxford and Manchester (Table E4; available online at https://doi.org/10.1016/j.ijrobp.2019.03.015). Additionally, in 20 of these samples there was an increase in the variation of staining intensities between tumors. Therefore, at Oxford attempts were made to improve the automated IHC staining on the BOND instrument to replicate that on the Dako processor. This involved adding a preprimary antibody protein blocking step, increasing the primary antibody dilution to 1:6000, and reducing the primary antibody incubation time (see Methods and Materials for details; final SOP in Material E1, available online at https://doi.org/10.1016/j.ijrobp.2019.03.015). After reoptimization, staining was repeated on 116 TMA samples from BCON (Table 3). The reoptimized staining protocol resulted in moderate to high agreement between scorers (Fig. 2c, Table 1).

**Table 3:** Baseline characteristics of the 116 patients in the BCON tissue microarray dataset

Characteristics	n (%) or median (range)
Median age (range), y	75.65 (51.5-90.5)
Sex	
Male	101 (87.07)
Female	15 (12.93)
Tumor stage	
T1	5 (4.31)
T2	83 (71.55)
T3	23 (19.83)
T4a	5 (4.31)
Tumor:stromal ratio	
High	109 (96.46)
Low	4 (3.54)
Growth margins	
Broad	4 (3.54)
Infiltrative	109 (96.46)
Growth patterns	
Both	47 (41.59)
Papillary	10 (8.85)
Solid	56 (49.56)
Necrosis	
No	47 (41.59)
Yes	66 (58.41)

**Fig. 3:**
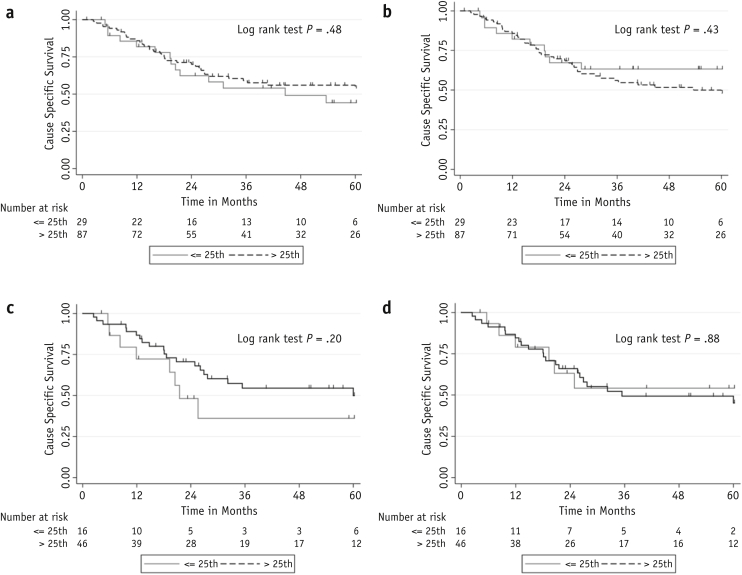
Kaplan-Meier survival plots for MRE11 expression >25th percentile or ≤25th percentile for (a) Oxford whole cohort; (b) Manchester whole cohort; (c) Oxford radiation therapy alone subgroup; and (d) Manchester radiation therapy alone subgroup BCON tissue microarrays.

In the 116 BCON TMA patients, there was no significant association between MRE11 expression and CSS (Fig. 3a and 3b). We hypothesized that the use of carbogen and nicotinamide could have influenced the response to RT via hypoxia modification. Therefore, a subgroup analysis of the 62 patients who received RT was performed. This analysis displayed a nonsignificant trend for an association with CSS when expression was scored in Oxford (log rank test, *P* = .20) but was not seen in scores from Manchester (Fig. 3c and 3d).

## Discussion

This study aimed to develop the MRE11 IHC assay for prospective clinical use. It was hoped the work would underpin the development of a trial randomizing patients to conventional versus MRE11-guided patient choice and subsequent introduction of routine MRE11 testing in the National Health Service. IHC is an attractive platform for clinical use, as illustrated by human epidermal growth factor receptor 2 testing,^13^ but routine implementation requires rigorous validation of the staining and scoring methods to ensure consistency and reliability across institutions.^14^ Automated staining aids standardization and efficiency by improving fidelity and workflow.^15^

We present the first attempt to validate MRE11 IHC staining across centers using good clinical laboratory practice standards. This was a large collaborative effort, and we provide a robust level of validation. The findings highlight the challenges associated with standardizing an MRE11 IHC test. Staining was qualitatively reproducible between centers, but scoring was not. Although histopathologists did not score the samples, they provided training, input on interpretation, and arbitration on challenging cases. Problems with interobserver scoring agreement were highlighted by the Ki67 Working Group,^16^ where scoring in 22 laboratories in 11 countries yielded ICC values ranging from 0.84 to 0.93. Our ICC values for density scores were similar, ranging from 0.90 to 0.98, but there were discrepancies in intensity scores despite efforts to improve agreement between laboratories. Potential reasons for the poor concordance include insufficient training, potential subjective bias, or technical factors such as differences in screen resolution.

Scores generated by a single scorer in Birmingham (using the scoring cards generated, but with less intensive training) were more discordant than those generated in Oxford and Manchester, reflecting the need for external quality assessment schemes. Although training can improve levels of concordance (eg, for EGFR staining^17^) some stains are intrinsically more difficult to score than others,^18^ including MRE11. Another issue is time taken for scoring, with the Leeds Consultant Histopathologist taking 25 to 30 minutes per case (see Material E1; available online at https://doi.org/10.1016/j.ijrobp.2019.03.015). Automated digital image analysis might remove human scoring bias and allow rapid scoring of multiple samples.^19^ We attempted to score a subset of samples using automated digital analysis but found that when optimized to higher-intensity samples, the results for low-intensity samples were inaccurate, and vice versa. Therefore, we did not pursue this. The development of more sophisticated algorithms might resolve this problem.

We failed to validate the previous findings by ourselves and others.^6^, ^8^ Although this could be due to lack of biological effect, we think it is more likely the result of methodological issues, including problems in standardizing the automated staining and poor scoring reproducibility across centers because of difficulties in standardizing intensity scoring. Others found similar difficulties when studying ERCC1 expression using an 8F1 antibody in a large sample set from 2 phase 3 trials of adjuvant cisplatin in lung cancer. A change in the batch of antibody used resulted in an inability to validate the predictive effect of ERCC1 immunostaining.^20^ From a biological point of view, it appears paradoxical that high expression of a DNA damage signaling protein (MRE11) might be associated with better outcomes after a DNA damaging agent (ionizing radiation). However, we recently observed a truncated version of MRE11 in a bladder cancer cell line, which is still detected by the antibody used in this study,^21^ and we hypothesize that this might act in a dominant-negative fashion. This hypothesis is currently under investigation in a separate study.

With the reoptimized staining method, in light of sample depletion, we only obtained MRE11 data on 116 BCON patients, which provided only 82% power to detect a change in 3-year CSS between 43% and 70% between 2 MRE11 groups, as reported previously.^7^ A nonsignificant trend for a difference in CSS was seen in patients receiving RT alone, but only in the samples stained and scored in Oxford. Carbogen and nicotinamide are given to reduce hypoxia within tumors and, by increasing the biological effectiveness of RT,^22^, ^23^ can modify the association between MRE11 and CSS. With small numbers of patients, the RT-alone subgroup analysis was underpowered.

**Fig. E5:**
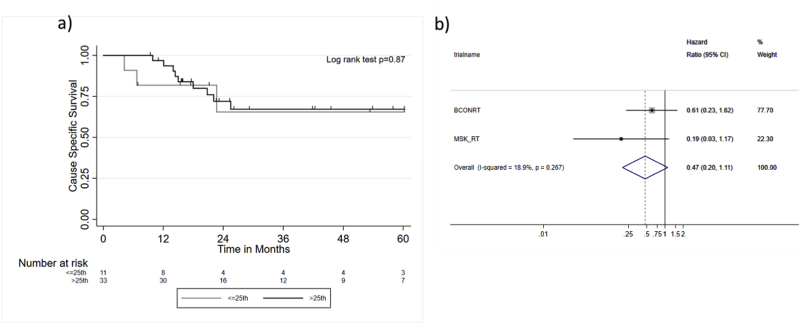


Despite our failure to validate MRE11 as a prognostic marker in RT patients, we cannot reject a role for MRE11 as a biomarker in MIBC. Indeed, a meta-analysis of the BCON RT alone with data obtained from 44 patients receiving bladder chemoradiation from Memorial Sloan Kettering (see Materials E1 for methods and results, Fig. E5; available online at https://doi.org/10.1016/j.ijrobp.2019.03.015) yielded a pooled hazard ratio for MRE11 >25th percentile of 0.47 (95% confidence interval, 0.13-1.03). This hazard ratio is similar to those reported previously (0.42 and 0.64).^7^, ^8^ Furthermore, recently the Radiation Therapy Oncology Group has taken an alternative approach to scoring MRE11 using an internal control of the nuclear:cytoplasmic ratio of MRE11 and the more standardizable automated quantitative analysis approach^24^ with promising results. Indeed, other DNA damage response genes have proved more tractable. For example, a recent microscopy-based nucleotide excision repair assay to profile *ERCC2* mutations established a role for *ERCC2* helicase domain mutations as a predictive biomarker in bladder cancer treated with cisplatin-based chemotherapy. *ERCC2* mutational status has now been incorporated as a predictive biomarker in risk-adapted MIBC clinical trials.^25^

Limitations of our study include the eventual reduced statistical power resulting from sample attrition and possibly use of TMAs. However, studies have identified concordance between IHC scoring of ≥0.6 mm TMAs and whole tissue sections, especially with multiple same-patient cores.^26^, ^27^ It is therefore reasonable to assume comparable results between the BCON whole tissue sections and 1 mm TMAs used here. In our study, death from other causes was treated as censored in the analysis. We attempted to apply competing risk analyses that take into account death from other causes. However, these resulted in findings similar to the analyses presented, and only 4 of 44 people in the Memorial Sloan Kettering cohort died from other causes. Therefore, we chose not to formally present competing risk analyses.

Numerous studies have identified potential IHC-based biomarkers, but only a few have obtained United States Food and Drug Administration approval.^14^ Despite the reduced stability of RNA versus protein, it is easier to measure at low abundance and with greater sensitivity and specificity.^28^ Generation of a gene signature that reflects MRE11 protein expression might provide a more robust biomarker than IHC. Further exploration of a gene classifier would be worthwhile.

## Conclusions

In this study, we were unable to validate MRE11 as a robust, reproducible, predictive biomarker for RT response in MIBC. A large analysis of prospectively acquired tissue is required using the refined staining methodology, along with further exploration of automated digital scoring methods. Alternatively, biomarkers based on other proteins or genomic data may be better placed for clinical use in the future.

## Supplementary Data

Material E1

Table E1

Table E2

Table E3

Table E4
