# Supplementary material for: MRE11 as a Predictive Biomarker of Outcome After Radiation Therapy in Bladder Cancer
Source: Int J Radiat Oncol Biol Phys. 2019 Jul 15;104(4):809–18. doi: 10.1016/j.ijrobp.2019.03.015 (PMC6588678; doi:10.1016/j.ijrobp.2019.03.015)
Supplement: Material E1 [file mmc1.docx]

**SUPPLEMENTARY MATERIAL**

**Centres involved in the study**

The following work was undertaken in the respective centres:

1. Staining and scoring of BCON whole-mount slides: Oxford and Manchester

2. Staining of subset of BCON slides: Leeds

3. Staining and scoring of BC2001 slides: Oxford and Birmingham

4. Staining of BCON TMA slides: Oxford

5. Scoring of BCON TMA slides: Oxford and Manchester

6. BCON TMA construction: Manchester

7. Control in-house TMA construction: Oxford

8. Staining and scoring of cystectomy samples: Manchester.

**Materials and methods**

Tissue microarrays

Two in-house tissue microarrays (TMAs) containing 0.6 mm cores in diameter were created for assay development: (1) a ‘BIDD’ TMA comprising bladder cancer samples from Oxford, commercial tissues and FFPE cell pellets derived from bladder cell lines, selected to demonstrate different MRE11 staining intensities, and kidney barrier tissue; (2) a ‘commercial’ tissue TMA made from purchased FFPE bladder tumour blocks (Proteogenex Inc, CA), FFPE cell pellets from a bladder cancer cell line and kidney barrier tissue.

Memorial Sloan Kettering (MSK) data analysis

MSK data were collected under institutional review board-approved protocols (1). Data was obtained from 44 patients from who received chemoradiotherapy with 54-72 Gy and mainly gemcitabine or alternatively cisplatin or paclitaxel. Adjusted HR of MRE11 H-score >25^th^ quartile was calculated with adjustment for lymphovascular invasion, carcinoma in situ, hydronephrosis, neoadjuvant chemotherapy, gender, completion of TURBT and radiotherapy dose. A HR>1 would indicate a greater risk of CSS for MRE11 H-score >25^th^ percentile. The assumption of proportional hazard was checked using proportional hazard tests and Schoenfeld residuals. A meta-analysis of the association between Oxford MRE11 and CSS for patients receiving radiotherapy alone was performed using patients treated within the BCON study and patients from MSK.

**Results**

Leeds pathologist scoring

Fifty randomly-selected slides from this BC2001 test cohort stained in Oxford were scored for intensity and positive percentage in Leeds by a Consultant Histopathologist, looking at the whole slide to represent the routine NHS clinical setting. This assessment took 25 to 30 minutes per case.

MSK data analysis

The median H score for MSK was 200 (range 10-300). Figure S5a shows the Kaplan-Meier survival curve for MRE11 expression >25^th^ centile or =< 25^th^ centile for the MSK data and Figure S5b shows the results of a meta-analysis performed on the MSK data and the 62 BCON IHC RT alone patients analysed by Oxford. The adjusted pooled hazard ratio for high MRE11 was 0.47 with 95% CI (95% CI: 0.20, 1.11). The I^2^ of 19.1% indicates low heterogeneity between BCON Oxford and MSK studies, though most weight was given to the BCON Oxford data. The proportional hazard assumptions were met in both studies.

**MRE11 staining SOPs**

**IHC: MRE 11 staining using the Bondmax Autostainer (original version).**

1. PROCEDURE

The staining procedure is carried out as detailed in SOP 048, Leica Bondmax autostainer: use for immunohistochemistry.

MRE 11 protocol

- - 1. The following is set up *via* ‘**Add case’**

*Primary antibody:* 150 µl/slide

*Antigen retrieval*: ER1 for 20 min

*Dewax*: yes

*IHC Protocol*: FH1w:

1. Peroxide Block – 5 min
2. Bond wash solution – 0 min
3. Bond wash solution – 0 min
4. Bond wash solution – 0 min
5. MARKER – MRE11 – 15 min
6. Bond wash solution – 0 min
7. Bond wash solution – 0 min
8. Bond wash solution – 0 min
9. Post Primary – 8 min
10. Bond wash solution – 2 min
11. Bond wash solution – 2 min
12. Bond wash solution – 2 min
13. Polymer – 8 min
14. Bond wash solution – 2 min
15. Bond wash solution – 2 min
16. Deionized water – 0 min
17. Mixed DAB Refine – 0 min
18. Mixed DAB Refine – 10 min
19. Deionized water – 0 min
20. Deionized water – 0 min
21. Deionized water – 0 min
22. Hematoxylin – 1 min
23. Deionized water – 2 min
24. Bond wash solution – 2 min
25. Deionized water – 2 min

Practical procedure

- - 1. Dilute sufficient MRE11 primary antibody 1:3,000 in antibody diluent and dispense into an open or titration container and place the container in a reagent rack and the rack into the Bondmax along with the Bond Polymer Refine Detection Kit and the prepared slides for staining.
    2. Carry out the run as detailed in SOP 048, Leica Bondmax autostainer: use for immunohistochemistry.
    3. When complete, remove the slides, and dehydrate and mount as detailed below:-

1. 50% EtOH – 1 min
2. 70% EtOH – 1 min
3. 100% EtOH – 1 min
4. 100% EtOH – 1 min
5. Xylene I. – 1 min
6. Xylene II. – 1 min
7. Cover with DPX
8. Data Analysis

- Slides can be viewed on a standard light microscope or slide scanner and assessed/scored according to antibody specific protocols.
- Image capture will depend on the equipment used.

**IHC: MRE 11 staining using the Bondmax Autostainer (TMA version).**

1. PROCEDURE

The staining procedure is carried out as detailed in SOP 048, Leica Bondmax autostainer: use for immunohistochemistry.

MRE 11 protocol

- - 1. The following is set up *via* ‘**Add case’**

*Primary antibody:* 150 µl/slide

*Antigen retrieval*: ER1 for 20 min

*Dewax*: yes

*IHC Protocol*:

1. Peroxide Block – 5 min
2. Bond wash solution – 0 min
3. Bond wash solution – 0 min
4. Bond wash solution – 0 min
5. Pre-Primary Block- 10% Albumin Bovine Serum in PBS – 30 min
6. Bond wash solution – 0 min
7. Bond wash solution – 0 min
8. Bond wash solution – 0 min
9. MARKER – MRE11 – 8 min
10. Bond wash solution – 0 min
11. Bond wash solution – 0 min
12. Bond wash solution – 0 min
13. Post Primary – 8 min
14. Bond wash solution – 2 min
15. Bond wash solution – 2 min
16. Bond wash solution – 2 min
17. Polymer – 8 min
18. Bond wash solution – 2 min
19. Bond wash solution – 2 mins
20. Deionized water – 0 min
21. Mixed DAB Refine – 0 min
22. Mixed DAB Refine – 10 min
23. Deionized water – 0 min
24. Deionized water – 0 min
25. Deionized water – 0 min
26. Haematoxylin – 1 min
27. Deionized water – 2 min
28. Bond wash solution – 2 min
29. Deionized water – 2 min

Practical procedure

- - 1. Dilute sufficient MRE11 primary antibody 1:6,000 in 10% Albumin Bovine Serum in PBS and dispense into an open or titration container and place the container in a reagent rack and the rack into the Bondmax along with the Bond Polymer Refine Detection Kit and the prepared slides for staining.
    2. Carry out the run as detailed in SOP 048, Leica Bondmax autostainer: use for immunohistochemistry.
    3. When complete, remove the slides, and dehydrate and mount as detailed below:-

1. 50% EtOH – 1 min
2. 70% EtOH – 1 min
3. 100% EtOH – 1 min
4. 100% EtOH – 1 min
5. Xylene I. – 1 min
6. Xylene II. – 1 min
7. Cover with DPX
8. Data Analysis

- Slides can be viewed on a standard light microscope or slide scanner and assessed/scored according to antibody specific protocols.
- Image capture will depend on the equipment used.

**Supplementary Figure legends**

**Figure S1**: a) Range card for scoring; b) 1 mm TMA holes in invasive areas in BCON samples.

**Figure S2**: **Comparison of Bond autostainers and MRE11 antibodies** (same lot ordered separately) in Oxford and Manchester on commercial tissues and Mini Tissue TMA (x40).

**Figure S3**: Comparison of Oxford, Manchester and Leeds autostainers.

**Figure S4**: **Kaplan-Meier curves of cause-specific survival versus MRE11 SQS (>25 or <=25 centiles)** for a) BCON Oxford; b) BCON Manchester; c) BC2001 scored by Oxford; d) cystectomy cohort.

**Figure S5**: a) Kaplan-Meier survival plot for MRE11 expression >25^th^ centile or =< 25^th^ centile for MSK cohort; b) Forest plot for meta-analysis of MSK cohort and the BCON IHC RT alone patients.

**References**

1. Desai NB, Scott SN, Zabor EC, et al. Genomic characterization of response to chemoradiation in urothelial bladder cancer. Cancer. 2016;122(23):3715-23.
