# Supplementary material for: MRE11 as a Predictive Biomarker of Outcome After Radiation Therapy in Bladder Cancer
Source: Int J Radiat Oncol Biol Phys. 2019 Jul 15;104(4):809–18. doi: 10.1016/j.ijrobp.2019.03.015 (PMC6588678; doi:10.1016/j.ijrobp.2019.03.015)
Supplement: Table E1 [file mmc2.docx]

**Table S1: Summary of FFPE samples available for study.** *Kidney tissue cores were placed at the edges of the TMAs to form a barrier.

| Cohort studied | | Treatment | Number of samples | Study samples | Comments |
| --- | --- | --- | --- | --- | --- |
| BCON | Randomised trial | RT vs RT+CON | 132/333 |  | Blocks available for 213 samples |
| BC2001 | Randomised trial | RT vs RT+5FU+MitC | 287/428 |  |  |
| Cystectomy | Cystectomy cohort | Cystectomy | 100 |  |  |
| BCON TMA | Randomised trial | RT vs RT+CON | 116 |  |  |
| BIDD TMA | Patient bladder tumour samples, commercial bladder tumour tissues, bladder cancer cell line pellets, human tonsil | n/a | 23 | 12 cores from 12 patient samples, 10 cores from 5 commercial bladder samples, 12 cores from 7 cell lines, 2 cores from 1 tonsil sample* |  |
| Commercial tissue TMA | Commercial bladder tumour samples, bladder cancer cell line  pellets |  | 6 | 6 cores from 5 commercial bladder samples, 5 cores from 1 cell line* |  |
