# Supplementary material for: MRE11 as a Predictive Biomarker of Outcome After Radiation Therapy in Bladder Cancer
Source: Int J Radiat Oncol Biol Phys. 2019 Jul 15;104(4):809–18. doi: 10.1016/j.ijrobp.2019.03.015 (PMC6588678; doi:10.1016/j.ijrobp.2019.03.015)
Supplement: Table E2 [file mmc3.docx]

**Table S2**: Median H-scores and ranges of MRE11 intensity scores.

|  | Median H-score | Range |
| --- | --- | --- |
| Oxford BCON Initial | 197 | 56-300 |
| Manchester BCON Initial | 195 | 55-300 |
| Oxford BC2001 | 195 | 13-299 |
| Manchester BCON RE-optimised | 148 | 3-299 |
| Manchester BCON reoptimised | 149 | 4-286 |
| Cystectomy (N=99) | 170 | 10-359 |
