# Supplementary material for: MRE11 as a Predictive Biomarker of Outcome After Radiation Therapy in Bladder Cancer
Source: Int J Radiat Oncol Biol Phys. 2019 Jul 15;104(4):809–18. doi: 10.1016/j.ijrobp.2019.03.015 (PMC6588678; doi:10.1016/j.ijrobp.2019.03.015)
Supplement: Table E3 [file mmc4.docx]

**Table S3:** Comparison of scoring by Centre A and Centre B scores.
