# Supplementary material for: MRE11 as a Predictive Biomarker of Outcome After Radiation Therapy in Bladder Cancer
Source: Int J Radiat Oncol Biol Phys. 2019 Jul 15;104(4):809–18. doi: 10.1016/j.ijrobp.2019.03.015 (PMC6588678; doi:10.1016/j.ijrobp.2019.03.015)
Supplement: Table E4 [file mmc5.docx]

**Table S4:** Analysis of 20 BCON tumour samples stained in Leeds, Manchester and Oxford.

|  | Leeds | Manchester | Oxford |
| --- | --- | --- | --- |
| Intensity | 1.9 | 2 | 2.5 |
| Density | 90.10% | 96.90% | 94.10% |
| H-score | 172.7 | 208.5 | 231.7 |
